# Use of Preservative Agents and Antibiotics for Increased Poliovirus Survival on Positively Charged Filters

**DOI:** 10.1007/s12560-017-9306-4

**Published:** 2017-06-14

**Authors:** Christine Susan Fagnant, Alexandra Lynn Kossik, Nicolette Angela Zhou, Liliana Sánchez-Gonzalez, Jill Christin Falman, Erika Karen Keim, Yarrow Linden, Alana Scheibe, Kilala Sayisha Barnes, Nicola Koren Beck, David S. Boyle, John Scott Meschke

**Affiliations:** 1 Department of Environmental & Occupational Health Sciences, University of Washington, 4225 Roosevelt Way NE, Suite 100, Seattle, WA 98105 USA; 2 PATH, 2201 Westlake Avenue, Suite 200, Seattle, WA 98121 USA

**Keywords:** ViroCap filters, Environmental surveillance, Poliovirus, Filtration, Virus recovery, Preservatives

## Abstract

Environmental surveillance of poliovirus (PV) and other non-enveloped viruses can help identify silent circulation and is necessary to certify eradication. The bag-mediated filtration system is an efficient method to filter large volumes of environmental waters at field sites for monitoring the presence of viruses. As filters may require long transit times to off-site laboratories for processing, viral inactivation or overgrowth of bacteria and fungi can interfere with virus detection and quantification (Miki and Jacquet in Aquatic Microb Ecol 51(2):195–208, 2008). To evaluate virus survival over time on ViroCap^™^ filters, the filters were seeded with PV type 1 (PV1) and/or MS2 and then dosed with preservatives or antibiotics prior to storage and elution. These filters were stored at various temperatures and time periods, and then eluted for PV1 and MS2 recovery quantification. Filters dosed with the preservative combination of 2% sodium benzoate and 0.2% calcium propionate had increased virus survival over time when stored at 25 °C, compared to samples stored at 25 °C with no preservatives. While elution within 24 h of filtration is recommended, if storage or shipping is required then this preservative mixture can help preserve sample integrity. Addition of an antibiotic cocktail containing cephapirin, gentamicin, and Proclin^™^ 300 increased recovery after storage at 4 and 25 °C, when compared to storage with no antibiotics. The antibiotic cocktail can aid sample preservation if access to appropriate antibiotics storage is available and sample cold chain is unreliable. This study demonstrated that the use of preservatives or antibiotics is a simple, cost-effective method to improve virus detection from ViroCap cartridge filters over time.

## Introduction

Environmental surveillance of pathogenic viruses is crucial for monitoring the spread of disease. Poliovirus (PV) and the male-specific coliphage MS2 are used extensively as models for enteric viruses (Popat et al. 2010; Grabow 2001). PV is a pathogen of international public health concern that the World Health Organization (WHO) is seeking to eradicate through the Global Polio Eradication Initiative (World Health Organization 2016). While clinical surveillance is the gold standard for monitoring the distribution of PV, fewer than 1 in 100 infections result in acute symptoms (Hovi et al. 2012). Asymptomatically infected individuals shed the virus in their stool; in a study where participants received a monovalent oral polio vaccine, Sabin-like (SL) PV type 1 and SL PV type 3 were shed at a load of up to 10^7^ infectious units/day (10^4^ infectious units/g stool) (Lodder et al. 2012).

Consequently, environmental surveillance of wastewater or wastewater-impacted surface water can detect silent PV transmission (El Bassioni et al. 2003) and is necessary for PV eradication certification (World Health Organization 2015). MS2 is used as an indicator species for fecal contamination (Gerba et al. 2003; Adams 1959) and environmental surveillance of MS2 can indicate if a beach area is unsafe for swimming or shellfish harvesting (Sobsey et al. 1978; Cormier et al. 2014; Cole et al. 2003). Environmental surveillance of organisms, such as PV or other enteric viruses, can result in early outbreak detection and a timely vaccine campaign launch before clinical cases are reported. It can also provide a more accurate epidemiological landscape of the distribution of waterborne viral pathogens. Additionally, environmental surveillance can inform on human exposure potential through determining whether locations are subject to fecal contamination.

The bag-mediated filtration system (BMFS) is a novel method to increase the sensitivity of virus and bacteriophage detection in environmental waters (Fagnant et al. 2014). The current, widely used environmental sampling method for PV involves processing a 0.5 L grab sample by the two-phase concentration method (polyethylene glycol/dextran), followed by virus isolation in tissue culture and intratypic differentiation (World Health Organization 2015). In contrast, the BMFS allows filtration of up to 10 L of surface water or primary effluent in the field and an average of 5.0 L (minimum 2.9 L) of influent wastewater (Fagnant et al. 2014). By eluting viruses from filters and subsequent secondary concentration into 12 mL, a higher effective volume can be assayed using the BMFS (750–2500 mL, with 3–10 L filtered) (Fagnant et al. 2014), as compared to the two-phase method (150 mL) (World Health Organization 2015). This scaling results in increased environmental surveillance sensitivity.

It is possible to process large sample volumes with the BMFS because sample filtration is completed in the field. Previously, collection of large samples was typically impractical due to field to laboratory transportation requirements and high costs for shipping of heavy, potentially biohazardous samples under cold chain. Since the BMFS enables filtration at the field site, only the cartridge filters are transported to an off-site laboratory for analysis. Filters are comparatively very small and lightweight, making their transport simpler than that of large liquid samples. During shipment, however, the concentrated viruses may be inactivated and the sample may experience overgrowth by bacteria and fungi. This is especially a concern in warmer climates where ambient temperatures can frequently reach 40 °C in the summer season. Temperature-controlled reverse cold chain shipping can be prohibitively expensive; overnight shipping is not possible for some international shipments, and significant delays in customs may occur. Therefore, alternative ways to maintain virus survival in the filters during shipment are needed.

The objective of this study was to test methods that may remove or reduce the need for shipping filters using reverse cold chain and may prolong sample integrity during longer shipping periods. The use of preservatives, including many food grade preservatives, and an antibiotic cocktail were investigated, as they can inhibit microbial overgrowth during filter storage or shipment. Additionally, food grade preservatives are non-toxic, inexpensive, easily acquired, do not require temperature-controlled storage, and do not have significant disposal considerations. The efficacy of preservatives and an antibiotic cocktail on PV and MS2 survival on positively charged filters over time was investigated, and the results are described in this work.

## Methods

### Study Organisms and Enumeration

A stock of the vaccine strain of Poliovirus type 1 (PV1) was prepared by confluent lysis of buffalo green monkey kidney (BGMK) cell monolayers (Sobsey et al. 1978). PV1 was extracted with Vertrel^™^ XF (E. I. du Pont de Nemours and Company, Wilmington, DE, USA) and purified stocks were stored at −80 °C (Mendez et al. 2000). PV1 was enumerated using a previously described plaque assay on 95% confluent BGMK cells (Sobsey et al. 1978) that was modified to use an Avicel RC-581 NF (FMC Health and Nutrition, Philadelphia, PA, USA) overlay rather than agarose (Matrosovich et al. 2006). BGMK cells were provided by Daniel R. Dahling (United States Environmental Protection Agency). All assays were performed in triplicate using 200 µL aliquots of relevant dilutions in 1× phosphate buffered saline (PBS, pH 7.4) on 9.5 cm^2^ wells. Infected cells were incubated (37 °C, 5% CO_2_, 48 h), stained (2% crystal violet in 20% methanol), and plaques were counted for infectious virus enumeration. Counts over 25 PFU/well were excluded as too numerous to count.

A stock of bacteriophage MS2 (ATCC 15597-B1) was prepared by confluent lysis on *Escherichia coli* F-amp (ATCC 70081) followed by chloroform extraction. Infectious MS2 was enumerated by the previously described double agar layer method on *E. coli* F-amp host (Adams 1959). All assays were performed in duplicate using 100 µL aliquots of relevant dilutions in 1x PBS pH 7.4. Plates were incubated (37 °C, 18–20 h) and plaques were counted. Counts over 300 PFU/plate were excluded as too numerous to count.

### Filter Media

Commercially available ViroCap^™^ pleated cartridge filters in polypropylene disposable housings were used in this study (Scientific Methods Inc., Granger, Indiana, USA). The filter media contain alumina nanofibers (2 nm diameter, 0.3 µm long) woven into glass fibers, resulting in an average pore size of 2 µm (Karim et al. 2009). The filter measures 4.6 cm high by 7 cm pleated diameter, with a total surface area of 57.96 cm^2^. The hold-up volume of the housing was 175 mL.

### Preliminary Preservative Investigations

**Fig. 1:**
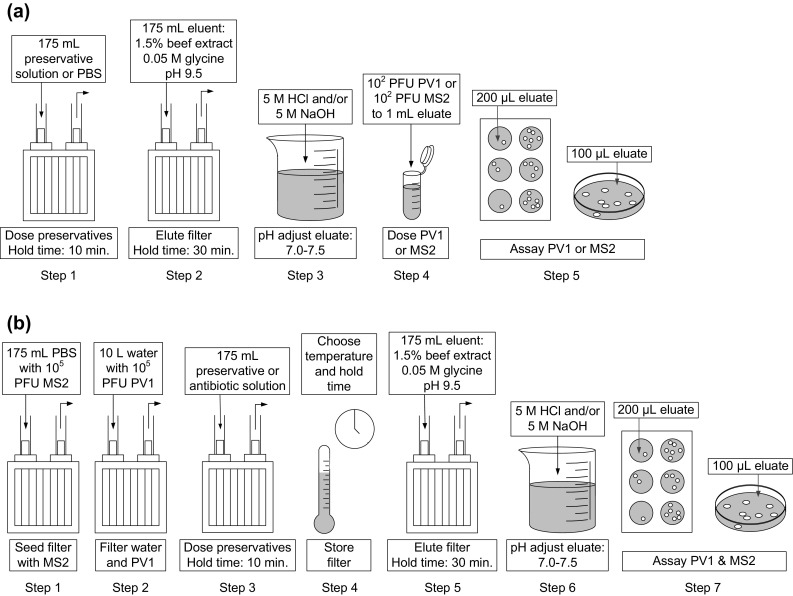
Workflow of **a** preliminary preservative and **b** preservative investigations. During the preliminary investigation, preservatives were dosed to the filter, the filter was eluted, and poliovirus type 1 (PV1) was added to the filter eluate. During the preservative investigations, however, PV1 was added to the water sample; the water sample was filtered; preservatives were dosed to the filter, and the filter was eluted

Preliminary investigations were completed to determine the effects of preservatives on PV1, MS2, BGMK, and *E. coli* F-amp survival. This was completed by passing 175 mL of preservative solution through the filter (Fig. 1a, Step 1), eluting the filter with 175 mL of sterile 1.5% beef extract (BD Diagnostics, Sparks, MD, USA), 0.05 M glycine, pH 9.5 (Fig. 1a, Step 2), and adjusting the pH of the eluate to 7.0–7.5 (Fig. 1a, Step 3). A 1 mL aliquot of the pH-adjusted eluate was spiked with ~10^2^ PFU PV1 (Fig. 1a, Step 4) and assayed for PV1 on BGMK cells (Fig. 1a, Step 5) or spiked with ~10^2^ PFU MS2 (Fig. 1a, Step 4) and assayed for MS2 using *E. coli* F-amp (Fig. 1a, Step 5). PBS controls were completed by passing 1× PBS through the filter instead of the preservative solution (Fig. 1a, Step 1).

Varying concentrations of four preservatives were prepared with deionized water and tested: (a) 0.005, 0.02, and 0.2% *o*-phenylphenol (Sigma Aldrich, St. Louis, MO, USA); (b) 0.1 and 2% calcium propionate (Acros Organics, Geel, Belgium) (pH 7.05 and 7.50, respectively); (c) 2% sodium benzoate (Sigma-Aldrich, St. Louis, MO, USA) (pH 7.34); (d) 0.02 and 0.04% sodium azide (ICN Biomedicals, Irvine, CA, USA); (e) 2% sodium benzoate and 0.2% calcium propionate (pH 7.55). These preservatives were chosen for their diversity in target organisms and in mechanisms of action (Luck and Jager 1997; Msagati 2012; Lichstein and Soule 1944). Duplicate filters were run for each condition and dilutions were plated in duplicate (undiluted, 1:10, and 1:100 dilutions in 1× PBS, pH 7.4 to aim for 1–2 PFU/cm^2^). PV1 or MS2 relative concentration was calculated by dividing the PV1 or MS2 measured in the sample by the PV1 or MS2 measured in the PBS control filter.

### Preservative Investigations

Preservative investigations were completed to determine the effect of different preservatives on PV1 and MS2 survival adsorbed on positively charged filters when stored at different temperatures and for different time periods.

#### Water

Ten-liter samples of influent wastewater were collected from West Point Treatment Plant (Seattle, WA, USA). Ten-liter samples of surface water were obtained from Lake Union (Seattle, WA, USA), a mesotrophic lake (City of Seattle 2010). Water was stored at room temperature and used within 7 days of collection. Water typically ranged in pH from 6.5 to 7.5.

#### Filtration

As an internal recovery control, approximately 10^5^ PFU of MS2 were seeded to 175 mL 1× PBS, vortexed (30 s), and passed through the filter (Fig. 1b, Step 1). Approximately 10^5^ PFU of PV1 were seeded to 10 mL 1× PBS, vortexed (30 s), and dosed to a 10 L water sample. The water sample was thoroughly mixed and passed through the MS2-seeded filter by a peristaltic pump (0.1–2.0 L/min; Fig. 1b, Step 2).

#### Preservative or Antibiotic Addition and Storage

After sample filtration with seeded PV1, 175 mL of a preservative solution or an antibiotic cocktail was added to the filter, left to stand 10 min, and then pumped out (Fig. 1b, Step 3). Filters were stored for 0, 3, or 7 days at 4, 25, 30, or 40 °C (Fig. 1b, Step 4). Samples held at 4 °C were stored in a refrigerator, and samples held at higher temperatures were stored in incubators. The preservatives evaluated included the following: 0.02% *o*-phenylphenol, 0.2% calcium propionate, 2% sodium benzoate, and a combination of 2% sodium benzoate and 0.2% calcium propionate. The antibiotic cocktail evaluated contained the following: 50 ppb cephapirin (LGC Standards, Manchester, NH, USA), 100 ppb gentamicin (Sigma Aldrich, St. Louis, MO, USA), and 15 ppm ProClin^™^ 300 (Supelco Inc., St. Louis, MO, USA). The effect of filter storage at different temperatures and storage times on PV1 recovery was also evaluated with no preservatives or antibiotics addition. Each condition was performed with 2–10 replicates.

#### Elution and Enumeration

Filters were eluted with 175 mL sterile 1.5% beef extract, 0.05 M glycine, pH 9.5 (Fig. 1b, Step 5). The eluent was added to the filter, left to stand 30 min, and then pumped out. After elution, the eluate was pH-adjusted to 7.0–7.5 using 5 M HCl and 5 M NaOH (Fig. 1b, Step 6). PV1 and MS2 samples were serially diluted in 1× PBS prior to analysis. Samples were plated within 2 h of elution (Fig. 1b, Step 7). Recovery was calculated by dividing the recovered count by the known seeded count.

#### Controls

Unseeded control volumes of 1× PBS, eluent, preservatives, antibiotic cocktail, and water matrix were plated onto BGMK cells (200 µL) and *E. coli* F-amp (100 µL) to ensure the absence of culturable enteric viruses and bacteriophages.

### Statistical Analyses

High degrees of variability in the composition of environmental samples may impact results. Because of this, the median absolute deviation (MAD) was used to determine outliers. This excludes

1 $$MAD = bM_{i} \left( {\left| {x_{i} - M_{j} \left( x \right)} \right|} \right),$$

where *b* is 1.4826 and *M*_i_ is the median of the absolute deviations from the median (*M*_j_) (Leys et al. 2013).

Unpaired Student’s or Welch’s *t*-tests were used to compare recoveries under different conditions.

## Results and Discussion

### Preliminary Preservative Investigations

**Fig. 2:**
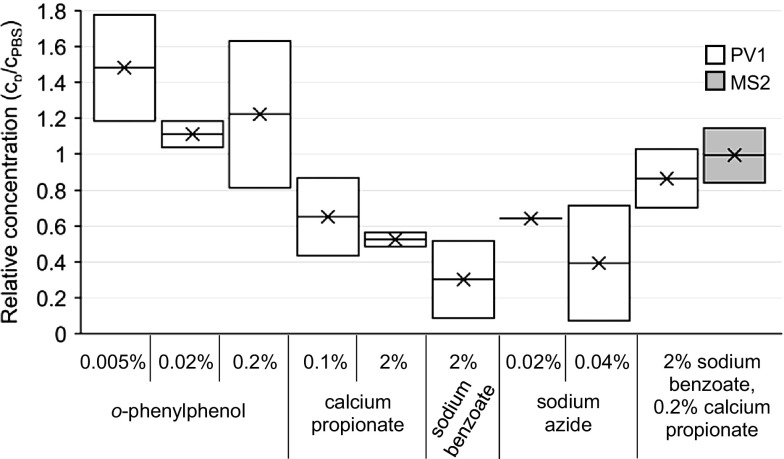
Relative concentration of PV1 (*white*) and MS2 (*gray*) spiked into eluate from filters dosed with preservatives. Relative concentration was determined by dividing the PV1 or MS2 measured from filters dosed with preservatives by PV1 or MS2 measured in control filters (PBS dosed only). Box and whisker plot:* upper*,* middle*, and* lower box lines* show the first, second, and third quartiles, respectively; markers ‘*x*’ show the mean (*n* = 2) (Color figure online)

To determine the effects of preservatives on tissue culture and PV survival, preservatives were passed through the filters with a hold time of 10 min, the filter was eluted with an eluent hold time of 30 min, and then the eluate was spiked with PV1 or MS2 (Fig. 1a). By directly spiking the eluate recovered from preservative-dosed filters with PV1 or MS2, the effects of the preservatives within the beef extract matrix were simulated and the variability due to recovering viruses from filters was removed. This focused the analysis on potential inhibition or cytotoxicity caused by the agent of interest. The amount of PV1 detected in the different eluates (obtained from filters dosed with four different preservatives at varying concentrations) was divided by the amount of PV1 detected in the eluates obtained from control filters (PBS only) (Fig. 2).

Three concentrations of *o*-phenylphenol were investigated (Fig. 2). *o*-Phenylphenol is a bacteriostatic and fungistatic food preservative that is used on the surface of citrus fruits (Smith 2011) and a rise in pH enhances the preservative effects (Luck and Jager 1997). It denatures the cell wall, inhibits carotin synthesis, and inhibits the NAD-oxidase enzyme system (Luck and Jager 1997). With 0.005% *o*-phenylphenol, the relative PV1 concentration was 1.48x (*n* = 2), which may be due to disaggregation of PV1 from exposure to the chemical. Relative PV1 concentration was not statistically different with the addition of 0.02% *o*-phenylphenol when compared to the relative PV1 concentration with the addition of 0.2% *o*-phenylphenol [1.11× (*n* = 2) and 1.22× (*n* = 2), respectively] (*p* = 0.83, *t* test). However, interpretation of the results for 0.2% *o*-phenylphenol samples was complicated by cytotoxicity. Cytotoxicity was observed in BGMK cells for most undiluted and 10^−1^ dilution samples, resulting in unreliable data. Therefore, a concentration of 0.02% was chosen for further investigations as it provided the highest protection against bacterial and fungal growth while minimizing cytotoxicity.

Two concentrations of calcium propionate were also investigated (Fig. 2). Calcium propionate is a fungistatic and bacteriostatic food preservative that is primarily used in baked goods (Belz et al. 2012), and is most effective at a basic pH (Luck and Jager 1997). It inhibits growth of fungi and bacteria by accumulating inside the cell and decreasing the pH of the cytosol (Luck and Jager 1997). An added benefit of calcium propionate is that it is chemically stable at room temperature. With 0.1% calcium propionate, the relative PV1 concentration was 0.65× (*n* = 2), while with a 2% concentration, the relative PV1 concentration was 0.52× (*n* = 2). To maximize PV growth and calcium propionate antimicrobial activity, a 0.2% calcium propionate concentration was chosen for future investigations.

Sodium benzoate was tested for its effect on PV1 recovery (Fig. 2). It is a bacteriostatic and fungistatic preservative agent used in jams and soda drinks (Msagati 2012). It is most effective when available in its unprotonated form at an acidic pH, because it becomes lipid-soluble and can penetrate the cell wall (Luck and Jager 1997; Msagati 2012). It works by accumulating in the cell, decreasing the pH, and inhibiting cell metabolic functions such as acetic acid metabolism and oxidative phosphorylation (Luck and Jager 1997; Msagati 2012). Like calcium propionate, sodium benzoate is stable at room temperature. A concentration of 2% sodium benzoate was tested, with a relative PV1 concentration of 0.30× (*n* = 2) and no cell cytotoxicity. This 2% concentration was used in further experiments.

Sodium azide was tested for its effect on PV1 recovery at two concentrations (Fig. 2). It is a bacteriostatic chemical effective at all pH levels. While not a food preservative, sodium azide is inhibitory to most gram-negative bacteria, some gram-positive bacteria, and yeasts (Kasimova et al. 2014; Rikhvanov et al. 2002). Similar to cyanide, it inhibits the activity of cytochrome c oxidase (Kasimova et al. 2014; Rikhvanov et al. 2002). With both 0.02 and 0.04% sodium azide concentrations, the PV1 growth in tissue culture was significantly lower than the control (*p* = 0.02, *t*-test), with aide 0.64× (*n* = 2) and 0.39× (*n* = 2), respectively. Also, sodium azide is acutely toxic on the human central nervous system, lowers the blood pressure, has mutagenic effects, and presents significant disposal considerations (Luck and Jager 1997). For these reasons, sodium azide was eliminated from further investigations.

A combination of 2% sodium benzoate and 0.2% calcium propionate was assessed for its effect on PV1 and MS2 recovery (Fig. 2), as sodium benzoate inhibits bacteria and is most effective at a low pH (Luck and Jager 1997), while calcium propionate inhibits fungi and is most effective at a higher pH (Luck and Jager 1997; Msagati 2012). This combination resulted in 0.87× (*n* = 2) relative PV1 concentration and 0.99× (*n* = 2) relative MS2 concentration when compared to the PBS control.

### Preservative Investigations

#### Influent Wastewater

Preservative investigations were conducted by dosing MS2 onto the filters, filtering 10 L volumes of influent wastewater spiked with PV1, adding preservatives or antibiotics to the filters and then pumping them out, and finally storing filters for specified temperatures and time periods before elution.

##### PV1

**Fig. 3:**
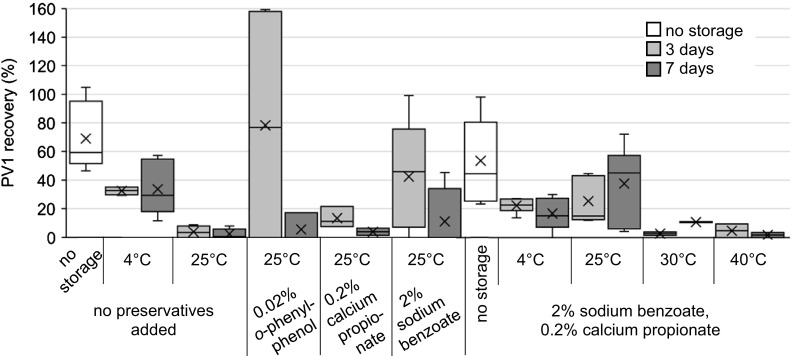
Recovery of PV1 dosed into influent wastewater and filtered through ViroCap filters followed by filter storage with preservatives. Samples were eluted from the filters after the indicated time.* Box* and* whisker plot*: *upper*, *middle*, and *lower box lines* show the first, second, and third quartiles, respectively;* whiskers* show the minimum and maximum data points; markers ‘*x*’ show the mean

**Fig. 4:**
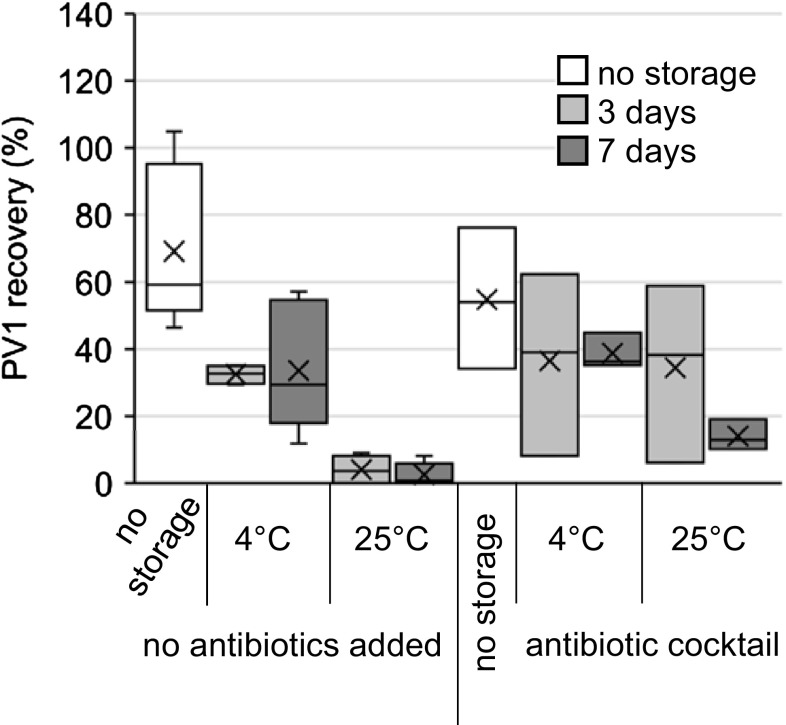
Recovery of PV1 dosed into influent wastewater and filtered through ViroCap filters followed by filter storage with an antibiotic cocktail (cephapirin, gentamicin, and ProClin 300). Samples were eluted after the indicated time.* Box* and* whisker plot*: *upper*, *middle*, and *lower box lines* show the first, second, and third quartiles, respectively; whiskers show the minimum and maximum data points; markers ‘*x*’ show the mean

The survival of PV1 on filters over time when spiked into influent wastewater was tested (Figs. 3, 4; Table 1). PV1 recovery with no preservatives added and no storage of the filter (filter processed same day), averaged 69.2% (Fig. 3; Table 1). PV1 recovery decreased after storage at 4 °C (*p* = 0.005, *t*-test) and 25 °C (*p* = 0.0005, *t*-test). Water samples were collected on different days across summer, autumn, and winter. Variability in recovery may be attributed to the differences in the specific biological composition of water samples, which changes with seasonal temperatures (Park et al. 2015).

**Table 1:** Poliovirus type 1 (PV1) and MS2 recovery from ViroCap filters after storage with the addition of preservatives or antibiotics

Additive	Storage temperature (°C)	Storage time (days)	PV1		MS2	
			Average recovery (%)	*N*	Average recovery (%)	*N*
Influent wastewater						
N/A	N/A	N/A	69.2	6	nt	nt
	4	3	32.5	4	nt	nt
	4	7	33.6	6	nt	nt
	25	3	4	6	nt	nt
	25	7	2.5	5	nt	nt
0.02% *o*-Phenylphenol	25	3	78.3	4	nt	nt
	25	7	5.7	3	nt	nt
0.2% Calcium propionate	25	3	13.5	3	nt	nt
	25	7	4.1	2	nt	nt
2% Sodium benzoate	25	3	42.4	5	nt	nt
	25	7	11.3	4	nt	nt
2% Sodium benzoate and 0.2% calcium propionate	N/A	N/A	53.5	10	84.6	9
	4	3	22.1	6	113.1	2
	4	7	16.7	7	48.6	2
	25	3	25.2	5	48.8	3
	25	7	37.6	6	10.5	5
	30	3	2.8	2	25.7	2
	30	7	10.8	2	2.5	2
	40	3	4.8	2	0.6	2
	40	7	1.8	2	nd	2
Antibiotics	N/A	N/A	54.3	3	96.6	3
	4	3	36.5	3	42.8	2
	4	7	38.8	3	18.6	2
	25	3	34.5	3	25.3	3
	25	7	14	3	6.4	2
Lake water						
N/A	N/A	N/A	37.8	6	nt	nt
2% Sodium benzoate and 0.2% calcium propionate	N/A	N/A	25	3	26.4	2
	4	3	14.7	2	11	3
	4	7	10	3	12.7	3
	25	3	14.1	3	1.7	2
	25	7	3	3	nd	3

*N*/*A* not applicable, *nt* not tested, *nd* not detected

PV1 recovery from filters dosed with *o*-phenylphenol, calcium propionate, sodium benzoate, and a combination of calcium propionate and sodium benzoate were determined after storage for 3 or 7 days (Fig. 3; Table 1). Filters dosed with 0.02% *o*-phenylphenol and stored at 25 °C had an average recovery of 78.3% after 3 days and 5.7% after 7 days. However, significant cytotoxicity was observed on the BGMK cells with these samples, and plaques were only readable at a 10^−2^ sample dilution, resulting in low sensitivity and high capacity for error. Consequently, *o*-phenylphenol was not used in future experiments. Filters treated with 0.2% calcium propionate and stored at 25 °C averaged 13.5 and 4.1% recovery after 3 and 7 days, respectively. Filters treated with 2% sodium benzoate and stored at 25 °C averaged 42.4 and 11.3% recovery after 3 and 7 days, respectively.

A combination of sodium benzoate and calcium propionate was tested (Fig. 3; Table 1), and with the addition of the preservative cocktail and no sample storage, PV1 recovery averaged 53.5%. This was lower than the PV1 recovery with no preservative addition and no storage (69.2%), although the difference in recoveries was not statistically significant (*p* = 0.14, *t*-test). Storage in 2% sodium benzoate and 0.2% calcium propionate at 4 °C resulted in PV1 recoveries that were lower than the PV1 recoveries in the 4 °C control (Fig. 3; Table 1) (*p* = 0.008, *t*-test). However, when taking into account the effect of preservatives on PV1 recovery, the recoveries after storage at 4 °C with and without preservatives added were not statistically different (*p* = 0.08, *t*-test). For storage at 25 °C, the addition of the preservatives resulted in a higher PV1 recovery (25.2 and 37.6% after 3 and 7 days, respectively) than the 25 °C control (4.0 and 2.5% after 3 and 7 days, respectively) (*p* = 0.0009, *t*-test). Also, PV1 recovery with the preservative combination is higher after storage at 25 °C than after storage at 4 °C, though the results are not statistically significant (*p* = 0.052, *t*-test). This is likely due to an uptake of the preservatives at 25 °C that is not present at 4 °C due to reduced biological activity. Because ambient temperatures can reach 40 °C during hot months in many field sampling areas, PV1 recoveries were determined after storage at 30 and 40 °C with the preservative cocktail added. Filters stored at 30 °C had a PV1 recovery of 2.8% after 3 days and 10.8% after 7 days. The increase in recovery after 7 days is likely due to sample variability, as only two replicates were evaluated. The large decrease in PV1 recoveries from storage at 25 °C to storage at 30 °C (*p* = 0.002, *t*-test) was likely due to antagonistic microflora (Sobsey and Meschke 2003). PV1 recoveries with storage at 40 °C were even lower and these low virus recoveries suggest that PV1 is unstable when stored at elevated temperatures, a result already observed in past studies (Sobsey and Meschke 2003; U.S. Environmental Protection Agency 2003).

These data indicate that with no preservatives added, biological activity in ViroCap filters is inhibited by refrigeration, while PV1 survival decreases significantly at ambient temperature. With the addition of 2% sodium benzoate and 0.2% calcium propionate, however, PV1 recovery after storage at ambient temperature (25 °C) was not statistically different from PV1 recovery with no added preservative and storage at 4 °C (*p* = 0.4, *t*-test). While the preservative agents had a negative impact on PV1 recovery with storage at 4 °C when compared to storage with no preservatives at 4 °C (*p* = 0.009, *t*-test), the preservatives had a positive impact when stored at 25 °C when compared to storage with no preservatives at 25 °C (*p* = 0.0009, *t*-test). Therefore, if reliable refrigeration is not possible due to limitations, shipping requirements, or other logistical factors, then addition of 2% sodium benzoate and 0.2% calcium propionate can prolong virus survival for up to 7 days when maintained at 25 °C. Sodium benzoate and calcium propionate chemicals require no special storage considerations, making them a particularly good option for field areas with minimal access to infrastructure. The combined effect of the preservatives at inhibiting both bacterial and fungal growth may result in increased viral survival, as captured the bacteria and fungi would be less likely to overgrow, graze upon, and inactivate captured viruses (Miki and Jacquet 2008). Additionally, because microbial activity generally increases at warm temperatures (e.g., 25–40 °C), the need for preservatives to inhibit microbial growth becomes more important under these conditions.

An antibiotic cocktail was also tested for its ability to preserve PV1 in influent wastewater (Fig. 4; Table 1). The antibiotics used (cephapirin, gentamicin, and ProClin 300) target fungi, yeasts, and bacteria. Cephapirin contains the β-lactam sub-structure present in penicillin, and inhibits cell wall synthesis (Schukken et al. 2013; White et al. 1979). It is effective against gram-negative and gram-positive bacteria (Schukken et al. 2013; Swelum 2013). Gentamicin is effective against gram-negative bacteria by binding directly to the ribosomal RNA, causing translational inaccuracy and inhibiting ribosome translocation (Yoshizawa et al. 1998). ProClin 300 is used as a preservative in diagnostic reagents. It inhibits the growth of bacteria, fungi, and yeasts by penetrating the cell wall to inhibit enzymes in the Krebs cycle (Nagy et al. 2005). Cephapirin and gentamicin require storage at 4 °C, and ProClin 300 should be stored at room temperature in a corrosives cabinet.

This antibiotic cocktail improved PV1 recoveries after filter storage as compared to when no antibiotics were added (*p* = 0.03, *t*-test) (Fig. 4; Table 1). The initial recovery with no storage was 54.8%. PV1 recoveries with antibiotics added when stored at 4 °C (36.5 and 38.8% after 3 and 7 days, respectively) were slightly higher than PV1 recoveries without antibiotics or preservatives added when stored at 4 °C (32.5 and 33.6% after 3 and 7 days, respectively), though the difference was not statistically significant (*p* = 0.4 and *p* = 0.3 for 3 and 7 days, respectively, *t*-test). PV1 recovery with antibiotics added after storage at 25 °C for 3 days (34.5%) was also not statistically different from PV1 recoveries after storage at 4 °C with no antibiotic or preservative addition (*p* = 0.5, *t*-test). However, PV1 recovery with antibiotics added after storage at 25 °C was significantly higher than with no antibiotics added and storage at 25 °C (*p* = 0.03, *t*-test). Therefore, if sampling facilities have access to appropriate storage areas and shipping at 4 °C is feasible, but an increase in temperature is a concern due to shipping or storage times, the antibiotic cocktail is recommended to improve PV1 preservation over time. Similarly to addition of the preservatives, the combined mechanisms of this antibiotic cocktail (inhibition of bacterial, fungal, and yeast growth) may result in increased viral survival by reducing the likelihood of microbial overgrowth, and the subsequent inactivation of captured viruses (Miki and Jacquet 2008).

##### MS2

**Fig. 5:**
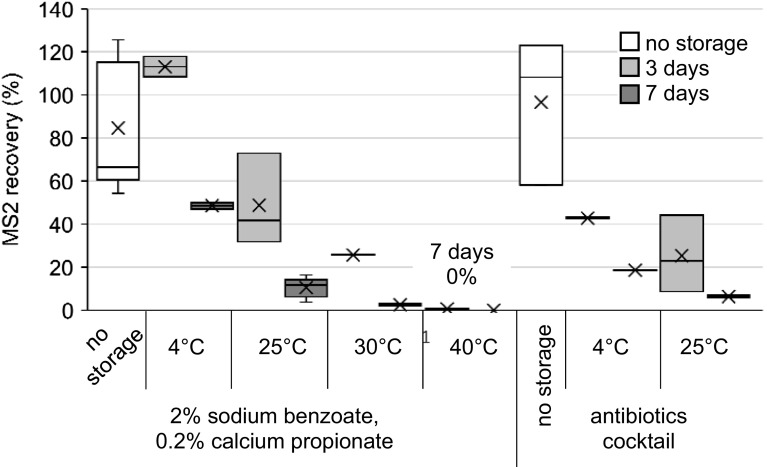
Recovery of MS2 after spiking onto ViroCap filters, filtration of influent wastewater, and filter storage with 2% sodium benzoate and 0.2% calcium propionate or an antibiotic cocktail (cephapirin, gentamicin, and ProClin 300). Samples were eluted after the indicated time.* Box* and* whisker plot*: *upper*, *middle*, and *lower box lines* show the first, second, and third quartiles, respectively; whiskers show the minimum and maximum data points; markers ‘*x*’ show the mean

MS2 is a nonpathogenic indicator organism for informing on fecal contamination (Cole et al. 2003), and has similar characteristics to enteric viruses (Grabow 2001). MS2 can be detected from waters using the ViroCap filter, and can also be used as a recovery control for ViroCap filters by pre-seeding the filter and analyzing for MS2 post-filtration. Unexpectedly low MS2 recovery may indicate compromised sample integrity. MS2 recoveries were examined by seeding ViroCap filters with MS2, filtering 10 L influent wastewater, adding 2% sodium benzoate and 0.2% calcium propionate or the antibiotic cocktail, and finally assaying MS2 (Fig. 5; Table 1).

With the addition of 2% sodium benzoate and 0.2% calcium propionate, an increase in storage temperature as well as an increase in storage time resulted in decreased MS2 recovery (Fig. 5; Table 1). This differs from PV1 recovery results, which are higher with storage at 25 °C than with storage at 4 °C, though not statistically significant (*p* = 0.052, *t*-test). With the addition of 2% sodium benzoate and 0.2% calcium propionate and no storage of the filter, MS2 recovery was 84.6%. Storage at 4 °C for 3 days resulted in MS2 recovery of 113.1% (possibly due to disaggregation during processing), indicating that no significant losses may have occurred during the storage of the filter. However, after storage at 4 °C for 7 days, MS2 recovery was significantly less than with no storage of the filter (*p* = 0.003, *t*-test). With an increase in storage temperature, MS2 recovery decreased. For example, after storage at 25 °C, MS2 recovery was significantly less than after storage at 4 °C (*p* = 0.02, *t*-test). Also, MS2 recovery with storage at 30 °C was 25.7% after 3 days, but dropped to 2.5% after 7 days, and with storage at 40 °C, MS2 recovery was 0.6% after 3 days and undetectable after 7 days.

The antibiotic cocktail also improved MS2 recovery from the filters (Fig. 5; Table 1). MS2 recovery with addition of the antibiotic cocktail and no storage was 96.6%. As anticipated, MS2 recovery was significantly lower with storage than with no storage (*p* = 0.03, *t*-test; antibiotic cocktail added; all temperature and storage times tested). The antibiotic cocktail was less effective than the preservative mixture when stored at 4 °C (*p* = 0.02 and *p* = 0.02 after 3 and 7 days, respectively), with MS2 recoveries of 42.8 and 18.6% after 3 and 7 days, respectively. The antibiotic cocktail was also less effective than the preservative mixture when stored at 25 °C, though not statistically significant (*p* = 0.1 and *p* = 0.06 after 3 and 7 days, respectively), with MS2 recoveries of 25.3 and 6.4% after 3 and 7 days, respectively.

Generally, MS2 recovery was higher than PV1 recovery in influent wastewater (*p* = 0.02, *t*-test; 2% sodium benzoate, 0.2% calcium propionate added; all temperature and storage times tested), which may be due to inhibitors in the wastewater impacting the tissue culture detection method (Greening et al. 2002). While the 2 µm pore size of the ViroCap filter concentrates and partially purifies influent wastewater, inhibitors can still pass into the sample. These inhibitors can result in cytotoxicity in tissue culture used for PV1 quantification, making plaques difficult or impossible to count, thereby reducing quantification sensitivity (Green and Lewis 1995; Greening et al. 2002). The *E. coli* F-amp culture used for MS2 quantification, however, did not exhibit any visible signs of impact from the filter eluate. Purification of the filter eluate by Vertrel XF or chloroform extraction, magnetic bead separation, ultracentrifugation, or another method may help to remove inhibitors and increase PV1 recovery detection (Green and Lewis 1995).

#### Lake Water

**Fig. 6:**
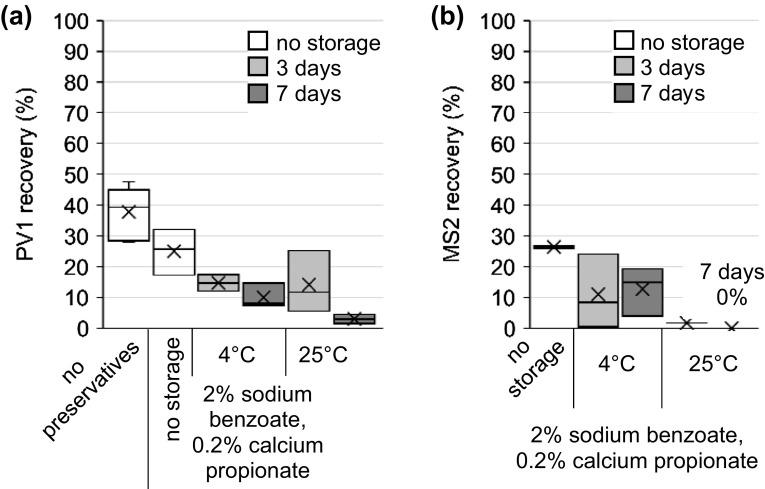
Recovery of **a** PV1 dosed into lake water and **b** MS2 spiked onto ViroCap filters followed by filtration of the lake water and filter storage with preservatives. Samples were eluted after the indicated time.* Box* and* whisker plot*: *upper*, *middle*, and *lower box lines* show the first, second, and third quartiles, respectively; whiskers show the minimum and maximum data points; markers ‘*x*’ show the mean

PV1 and MS2 were also analyzed for recovery from 10 L lake water (Fig. 6a, b; Table 1). With the addition of 2% sodium benzoate and 0.2% calcium propionate and no storage of the filter, initial recoveries for both PV1 (25.0%) (Fig. 6a) and MS2 (26.4%) (Fig. 6b) were lower with lake water than with influent wastewater [53.5% for PV1 (Fig. 3) and 84.6% for MS2 (Fig. 5)] (*p* = 0.008 and 0.0002 for PV1 and MS2, respectively, *t*-test). The greater initial PV1 and MS2 recovery in influent wastewater when compared to lake water may be due to the mechanism of virus retention in the ViroCap filter. PV and other viruses adsorb rapidly to particulate matter through a host of forces, including double-layer interactions and van der Waals attractions (Sobsey and Meschke 2003; Gerba and Bitton 1984). Particulates are then retained in the ViroCap filter along with associated adsorbed viruses, until release from the particulate matter through elution and recovery in the eluate (Nakajima et al. 2003). Additionally, adsorption to particulates can increase virus survival over time and at elevated temperatures (Nakajima et al. 2003).

When stored in 2% sodium benzoate and 0.2% calcium propionate, PV1 recovery after 3 days at 4 °C (14.7%) and at 25 °C (14.1%) was not statistically different from no storage with the preservative cocktail added (25.0%) (*p* = 0.07 and *p* = 0.1 for 4 °C and 25 °C, respectively, *t*-test) (Fig. 6a; Table 1). However, PV1 recoveries after 7 days at 4 °C (10.0%) and at 25 °C (3.0%) were lower when compared to PV1 recovery with no storage and with the preservative cocktail added (*p* = 0.03 and *p* = 0.02 for 4 °C and 25 °C, respectively, *t*-test) (Fig. 6a). The significant decrease in PV1 recovery from 3 days of storage to 7 days of storage [*p* = 0.04, *t*-test; all temperature tested (4 and 25 °C)] (Fig. 6a) is greater than the change in PV1 recovery from 3 to 7 days of storage with influent wastewater (*p* = 0.35, *t*-test; for 4 and 25 °C) (Fig. 3). This suggests that the virus attachment to particulate matter in the influent wastewater may offer a protective effect that is not present in the relatively clear lake water. Additionally, Lake Union fecal coliform levels have met water quality standards of less than 50 colony forming units (CFU) in 100 mL in 86% of samples between 1998 and 2005 (City of Seattle 2010), whereas raw wastewater typically contains fecal coliform concentrations ranging from 10^5^ to 10^8^ most probable number (MPN)/100 mL (Tchobanoglous et al. 2003), confirming the biological composition is significantly different between Lake Union and typical raw wastewater.

When stored in 2% sodium benzoate and 0.2% calcium propionate, MS2 recovery after 3 days (11.0%) and 7 days (12.7%) at 4 °C was lower than when compared to no storage with preservatives added (26.4%) (*p* = 0.006, *t*-test) (Fig. 6b; Table 1). However, there was no statistical difference between MS2 recovery when stored at 4 °C for 3 days and 7 days (*p* = 0.426, *t*-test). The relative stability of MS2 in filters over time when stored at 4 °C is likely due to the lower biological activity of mesotrophic lake water compared to influent wastewater, and the low recovery at 25 °C may be due to the lack of particulates in the lake water to provide a protective effect (Nakajima et al. 2003). Finally, MS2 recovery was very poor when stored at 25 °C with the preservative cocktail added, with a recovery of 1.7% after 3 days and not detectable after 7 days.

### Recommendations

Deciding how to store ViroCap filters to maximize PV1, MS2, and/or enteric virus detection is important. Water quality, cold chain reliability, and storage facilities all can impact sample integrity. For ViroCap filters containing water samples with few particulates, such as relatively clear surface water, storage at 4 °C may maximize virus recovery. However, additional data are necessary to determine if addition of preservatives with refrigeration results in higher recovery than simple refrigeration over time. For ViroCap filters containing water samples with high particulate and/or biological matter, such as wastewater, storage and/or shipping at 4 °C, is preferable, although not always feasible. If reliable refrigeration is not possible, addition of 2% sodium benzoate and 0.2% calcium propionate is recommended to maintain sample integrity by reducing bacterial and fungal overgrowth, and associated predation upon and inactivation of viruses. Addition of this preservative cocktail increases virus survival over time when ViroCap wastewater samples are stored up to 25 °C. In hot climates, shipping samples with a single ice pack may be sufficient to ensure samples remain at, or below 25 °C for several days in transit, and this is significantly more economical than shipping under conditions guaranteed to maintain refrigeration. Moreover, food grade preservatives are non-toxic in low concentrations, easy to dispose, inexpensive, and effective. Alternatively, cephapirin, gentamicin, and ProClin 300 can be added to reduce viral inactivation during shipping. However, these antibiotics require special storage (e.g., refrigeration or corrosives cabinet) and acquisition can be challenging. Addition of preservatives or antibiotics to cartridge filters after sampling wastewater presents a simple and cost-effective solution to virus preservation over time. In conjunction with BMFS, this method could open up new pathways for scientific research on environmental surveillance of viruses in locations without immediate access to laboratory facilities.
